# The intergenerational production of depression in South Korea: results from a cross-sectional study

**DOI:** 10.1186/s12939-016-0513-7

**Published:** 2017-01-13

**Authors:** B. G. Jeong, G. Veenstra

**Affiliations:** 1 Department of Preventive Medicine, School of Medicine & Institute of Health Science, Gyeongsang National University, 15, 816 Beon-Gil, Jinjudae-Ro, Jinju-si, Gyeongsangnam-Do 660-751 South Korea; 2 Department of Sociology, University of British Columbia, 6303 NW Marine Drive, Vancouver, BC V6T 1Z1 Canada

**Keywords:** South Korea, Depressive symptoms, Capitals, Intergenerational processes

## Abstract

**Background:**

Although a number of studies have uncovered relationships between parental capital and the manifestation of depression in their children, little is known about the mechanisms that undergird the relationships. This study investigates the intergenerational effects of the cultural and economic capitals of South Korean parents on depressive symptoms in their adult children and the degree to which the capitals of the adult children explain them.

**Methods:**

We employed nationally representative cross-sectional survey data from the 2006 Korea Welfare Panel Study. A sample of 11,576 adults over thirty years of age was used to investigate the intergenerational production of depression in South Korea. We applied binary logistic regression modelling to the Center for Epidemiological Studies Depression Scale (CES-D).

**Results:**

Parental education (institutionalized cultural capital) manifested an independent and statistically significant inverse association with depressive symptoms [OR = 1.680 (95% CI: 1.118-2.523) for men; OR = 2.146 (95% CI: 1484–3.102) for women]. Childhood economic circumstances (economic capital) had an independent and statistically significant inverse association with depressive symptoms among adult women only [OR = 2.009 (95% CI: 1.531-2.635)]. The education of the adult children themselves was strongly associated with depressive symptoms in the expected direction [OR = 4.202 (95% CI: 2.856-6.181) for men; OR = 4.058 (95% CI: 2.824-5.830)] and the most of the association between parental capitals and depressive symptoms was explained by the educational attainment of the children. Receipt of monetary inheritance from parents had a weak but statistically significant association with depression among men [OR = 1.248 (95% CI: 1.041-1.496)] but was unrelated to depression among women. A large portion of the association between respondent education and depressive symptoms was explained by household income. Finally, childhood economic circumstances were associated with depressive symptoms among women over and above the cultural and economic capitals held by the women themselves [OR = 1.608 (95% CI: 2.08-2.139)].

**Conclusions:**

Our study illuminates the importance of the intergenerational transmission of capitals for the development of depressive symptoms among adults in South Korea.

**Electronic supplementary material:**

The online version of this article (doi:10.1186/s12939-016-0513-7) contains supplementary material, which is available to authorized users.

## Background

Depression is a mood disorder characterized by overwhelmingly negative emotions and a sustained loss of pleasure [1–3]. At any given point in time, approximately one to three percent of the residents of industrialized countries experience persistent, recurring symptoms of depression [4, 5]. Depression can have a dramatic effect on people’s lives, impairing social, work and family functioning [6–8] and negatively affecting physical health and well-being [9].

Although a large body of research indicates that hereditary factors can predispose people to depression [10, 11], a growing body of evidence suggests that socioeconomic conditions are also important factors in the onset of depression. For instance, a number of studies have uncovered relationships between parental socioeconomic status and the manifestation of depression in their children [12, 13], associations that persist after controlling for parental depression [14, 15]. Other studies have determined that the risk of depression among adults is higher among people with lower levels of education, less prestigious occupations and lower incomes [16–18].

Children and adults living in poor socioeconomic circumstances typically experience more stressful living conditions [19], possess relatively low levels of control over their physical and social environments [20], face more difficulties creating intimate relationships [21] and experience poorer health than do people living in good socioeconomic circumstances [22, 23]. These factors can increase a person’s vulnerability to depression and reduce their ability to cope with it once manifested. In short, depression appears to be affected by socioeconomic circumstances at multiple stages of the life course.

However, the socioeconomic status of adults is shaped by the socioeconomic status of their parents [24–26], suggestive of a more complex causal storyline that runs from parental capitals to the capitals of the children and subsequently to the manifestation of depression in the children in adulthood. Capitals can be transmitted from parents to children through various processes. For instance, wealth can be directly transmitted to children by means of inheritance. Wealthier parents can also invest more money and time in educating their children. More highly educated parents have a greater capacity to develop linguistic and other cultural skills and talents in their children that can subsequently affect the latter’s performance in the education system and in the labor market. In other words, the capital resources possessed by parents can be transmitted to the next generation through a variety of processes that effectively serve to reproduce socioeconomic inequality across generations. Accordingly, depression in adults may be partly reflective of intergenerational capital transmission processes from parents to children.

The plausibility of this multigenerational line of causality notwithstanding, empirical studies that relate processes of intergenerational transmission of capital to adult health are rare [27, 28]. In particular, research on the relationship between processes of intergenerational capital transmission and depression, an important domain of mental health, is nonexistent. To address this gap, we use cross-sectional survey data from the 2006 Korean Welfare Panel Study to investigate the degree to which the capitals of South Korean parents are related to the capitals of their adult children and, ultimately, to the manifestation of depressive symptoms in them.

## Methods

### Data and study population

Our study uses data from the 2006 wave of the Korea Welfare Panel Study which has a response rate of 71.3%. We restricted our investigation to survey respondents (*n* = 12,292) who were thirty years of age or older at the time of the survey in order to produce stable measures of educational attainment. We further restricted our analyses to the 11,576 respondents (94.2% of the sample) who provided valid information for all of the variables used in our study. Table 1 describes characteristics of the latter sample.

**Table 1:** Characteristics of the survey sample (*n* = 11,576; un-weighted data)

Variable	Categories	Men		Women	
		n	%	n	%
Age	less than 40	1374	26.0	1430	22.7
	40 to 59	2095	39.6	2293	36.5
	60 and older	1819	34.4	2565	40.8
Marital status	married	4378	82.8	4294	68.3
	widowed	151	2.9	1442	22.9
	separated	52	1.0	82	1.3
	divorced	218	4.1	268	4.3
	single	489	9.3	202	3.2
Highest parental education	did not complete elementary school	2280	43.1	2932	46.6
	elementary school	1500	28.4	1617	25.7
	middle school	585	11.1	709	11.3
	high school	644	12.2	703	11.2
	technical college	56	1.1	75	1.2
	university	203	3.8	231	3.7
	graduate school	20	0.4	21	0.3
Economic conditions in childhood	very poor	566	10.7	581	9.2
	poor	1899	35.9	1914	30.4
	average	2228	42.1	2843	45.2
	rich	542	10.3	859	13.7
	very rich	53	1.0	91	1.5
Educational attainment	did not complete elementary school	420	7.9	1485	23.6
	elementary school	901	17.0	1373	21.8
	middle school	658	12.4	782	12.4
	high school	1792	33.9	1722	27.4
	technical college	407	7.7	298	4.7
	university	956	18.1	577	9.2
	graduate school	154	2.9	51	0.8
Received inheritance	yes	1452	27.5	960	15.3
	no	3836	72.5	5328	84.7
Household income	<1000	1205	22.8	1985	31.6
	1000-1999	1199	22.7	1418	22.6
	2000-2999	972	18.4	998	15.9
	3000-3999	680	12.9	654	10.4
	4000-4999	479	9.1	476	7.6
	5000-5999	246	4.7	246	3.9
	6000-6999	193	3.7	199	3.2
	7000-7999	139	2.6	127	2.0
	8000-8999	67	1.3	63	1.0
	9000-999	33	0.6	38	0.6
	>10000	75	1.4	84	1.3
Depressive symptoms	few (0–8 on the CES-D)	4154	78.6	4258	67.7
	regular (9 or more on the CES-D)	1134	21.4	2030	32.3

### Measures

#### Cultural capital

According to Bourdieu [29], cultural capital can exist in three forms. Cultural tastes and inclinations, a kind of *embodied* cultural capital, are lasting dispositions of mind and body. *Objectified* cultural capital refers to the possession of valued cultural goods. *Institutionalized* cultural capital is a “form of objectification which must be set apart because, as will be seen in the case of educational qualification, it confers entirely original properties on the cultural capital which it is presumed to guarantee” ([29], p. 248). In our study, respondents were asked about their mother and father’s levels of educational attainment, measures of institutionalized cultural capital from which we created a single variable assessing parental highest level of education. We also assessed the institutionalized cultural capital of the survey respondents themselves in the form of personal highest level of educational attainment. Both variables distinguished between seven levels of education: did not complete elementary school, completed elementary school, completed middle school, completed high school, completed technical college, completed university and completed graduate school. For use in regression modelling, we combined the elementary school and middle school categories for both variables and the university and graduate school categories for the parental education variable.

#### Economic capital

To assess parental economic capital, respondents were asked “What were your economic living conditions during childhood (0–17 years of age)?” with response categories ‘very poor,’ ‘poor,’ ‘average,’ ‘rich’ and ‘very rich.’ We combined the latter two categories for use in our regression analyses. The current household incomes of respondents were also calculated from a series of questions assessing income from multiple sources including employment, investments, pensions, social insurance, etc. Assessed in 10,000,000 won units, this right-skewed continuous variable ranged from a low of zero to a high of 30.8. A categorical version of household income is described in Table 1. Respondents were also asked “Have you ever received an inheritance or donation from your parents?” to which they could reply ‘yes’ or ‘no.’

#### Depression

Depression was measured by calculating the sum of 11 items derived from the Center for Epidemiological Studies Depression Scale (CES-D). The CES-D scale was developed to diagnose depression by way of assessing a range of depressive symptoms. This scale is a widely used measure of depression and several short CES-D forms have been developed from it [30, 31]. Each item was answered using a 4-point scale (0 = less than one day in the past week; 1 = two or three days in the past week; 2 = four or five days in the past week; 3 = six or seven days in the past week). Cronbach’s alpha for the 11 items from the CES-D scale in this sample was high at 0.88. Consistent with previous research [32–34], respondents were categorized into two groups: few depressive symptoms (72.7% of the sample scored 0–8 on the CES-D) and regular depressive symptoms (27.3% of the sample had a score of 9 or higher).

### Data analysis

We created a series of binary logistic regression models on depression separately for men (*n* = 5288) and women (*n* = 6288). These models are described in Tables 2 and 3. The first model in each table describes the effects of parental education and childhood economic circumstances on depression while controlling for age and marital status. The second model adds personal education to the first model, the third model adds receipt of inheritance money to the second model and the fourth model adds household income to the third model. This sequence of models enables us to identify the independent effects of parental cultural and economic capitals on depression and then investigate the degree to which various forms of capital held by the respondents potentially explain them. However, the problem of residual variance in logistic regression means that changes in regression coefficients across nested models can reflect changes in the scaling of the dependent variable [35]. Accordingly, we also applied the Karson/Holm/Breen (KHB) method of decomposing effects in non-linear probability models [36, 37] via the *khb* command in Stata [38] when investigating mediation. In all of our analyses we utilized the master weight variable provided with the Korea Welfare Panel Study to produce estimates that more closely represent the adult South Korean population.

**Table 2:** Binary logistic regression models on presence of depression in men (weighted data)

Variable	Categories	Model 1			Model 2			Model 3			Model 4		
		*OR*	*95% CI*		*OR*	*95% CI*		*OR*	*95% CI*		*OR*	*95% CI*	
Highest parental education	did not complete elementary school	1.680 *	1.118	2.523	1.150	0.754	1.755	1.147	0.752	1.750	1.004	0.653	1.545
	elementary or middle school	1.479 *	1.004	2.179	1.180	0.793	1.757	1.176	0.790	1.750	1.079	0.720	1.616
	high school	1.273	0.835	1.940	1.141	0.746	1.747	1.129	0.738	1.729	1.050	0.680	1.621
	technical college	1.772	0.873	3.597	1.724	0.843	3.527	1.751	0.854	3.590	1.773	0.852	3.691
	university (reference)	1.000			1.000			1.000			1.000		
Economic conditions in childhood	very poor	1.196	0.867	1.650	0.932	0.669	1.298	0.893	0.639	1.247	0.946	0.673	1.329
	poor	1.035	0.801	1.337	0.906	0.698	1.176	0.874	0.672	1.137	0.924	0.707	1.209
	average	0.863	0.672	1.109	0.836	0.649	1.076	0.823	0.639	1.060	0.845	0.653	1.093
	rich or very rich (reference)	1.000			1.000			1.000			1.000		
Educational attainment	did not complete elementary school				4.202 ***	2.856	6.181	4.165 ***	2.832	6.126	2.356 ***	1.584	3.502
	elementary or middle school				2.655 ***	2.048	3.441	2.634 ***	2.033	3.414	1.670 ***	1.274	2.190
	high school				1.583 ***	1.269	1.974	1.571 ***	1.259	1.960	1.171	0.932	1.471
	technical college				1.649 **	1.211	2.245	1.650 **	1.212	2.246	1.312	0.957	1.797
	university (reference)				1.000			1.000			1.000		
Received inheritance	no							1.248 *	1.041	1.496	1.270 *	1.056	1.526
	yes (reference)							1.000			1.000		
Household income	…										0.687 ***	0.644	0.734
Household income squared	…										1.013 ***	1.010	1.017
Hosmer and Lemeshow test		χ2 = 7.087 (p = 0.527)			χ2 = 4.268 (p = 0.832)			χ2 = 10.281 (p = 0.246)			χ2 = 27.638 (p = 0.001)		
−2 log likelihood		4597.925			4524.390			4518.519			4367.795		

Each model controls for age, square of age, and marital status. *N* = 5288 in each model. *p < 0.05, **p < 0.01, ***p < 0.001

**Table 3:** Binary logistic regression models on presence of depression in women (weighted data)

Variable	Categories	Model 1			Model 2			Model 3			Model 4		
		OR	95% CI		OR	95% CI		OR	95% CI		OR	95% CI	
Highest parental education	did not complete elementary school	2.146 ***	1.484	3.102	1.331	.901	1.967	1.324	.896	1.957	1.226	.824	1.824
	elementary or middle school	1.725 **	1.211	2.456	1.238	.856	1.791	1.232	.851	1.784	1.163	.798	1.694
	high school	1.710 **	1.172	2.494	1.378	.937	2.028	1.373	.933	2.022	1.319	.889	1.955
	technical college	1.165	.606	2.238	1.067	.549	2.073	1.065	.548	2.071	.906	.465	1.767
	university (reference)	1.000			1.000			1.000			1.000		
Economic conditions in childhood	very poor	2.009 ***	1.531	2.635	1.637 **	1.237	2.166	1.627 **	1.228	2.155	1.608 **	1.208	2.139
	poor	1.200	.979	1.470	1.059	.860	1.303	1.054	.855	1.298	1.134	.918	1.402
	average	.978	.807	1.186	.940	.774	1.141	.936	.770	1.137	.995	.816	1.214
	rich or very rich (reference)	1.000			1.000			1.000			1.000		
Educational attainment	did not complete elementary school				4.058 ***	2.824	5.830	4.031 ***	2.803	5.796	2.260 ***	1.551	3.293
	elementary or middle school				2.481 ***	1.844	3.339	2.468 ***	1.833	3.324	1.557 **	1.142	2.121
	high school				2.066 ***	1.607	2.656	2.059 ***	1.601	2.648	1.574 **	1.216	2.038
	technical college				1.056	.716	1.558	1.054	.714	1.555	.873	.589	1.295
	university (reference)				1.000			1.000			1.000		
Received inheritance	no							1.052	.875	1.266	1.052	.873	1.269
	yes (reference)							1.000			1.000		
Household income	…										.732 ***	.693	.774
Household income squared	…										1.010 ***	1.007	1.014
Hosmer and Lemeshow test		χ2 = 10.247 (p = 0.248)			χ2 = 14.232 (p = 0.076)			χ2 = 18.322 (p = 0.019)			χ2 = 12.817 (p = 0.118)		
−2 log likelihood		6029.167			5957.404			5957.110			5794.076		

Each model controls for age, square of age, and marital status. *N* = 6288 in each model. *p < 0.05, **p < 0.01, ***p < 0.001

## Results

Model 1 in Table 2 and Model 1 in Table 3 indicate that parental education manifests an independent and statistically significant association with depression among men and among women. Childhood economic conditions have an independent and statistically significant association with depression among women only.^1^

Model 2 in Table 2 and Model 2 in Table 3 indicate that personal education has a strong association with depression for both men and women. The declines in the effect sizes of parental education from Model 1 to Model 2 in both tables suggest that much of the association between parental resources and depression is explained by personal educational attainment. The KHB decomposition indicates that 73.1% of the association between parental education (most versus least educated) and depression is caught up in the educational attainment of the men; for women this percentage is 62.0%. A decline in the effect size of childhood economic conditions from Model 1 to Model 2 in Table 3 suggests that some of the association between childhood economic conditions and depression is explained by the educational attainment of the women; the KHB decomposition indicates that 30.1% of the association between childhood economic conditions (very poor versus rich or very rich) and depression is caught up in the educational attainment of the women (Additional file 1). However, childhood economic circumstances retain a statistically significant association with depression over and above the educational attainment of the respondents themselves, but only for women.

Model 3 of Table 2 indicates that receipt of inheritance from parents has a weak but statistically significant association with depression among men; inheritance is unrelated to depression among women. The fourth models in Tables 2 and 3 indicate that household income has a strong association with depression for both men and women, and the declines in effect size for respondent education from Model 3 to Model 4 in these tables suggest that some of the association between respondent education and depression is explained by respondent income. For men, the KHB decomposition indicates that 44.7% of the association between respondent education (most versus least educated) and depression is caught up in the incomes of the respondents; for women this percentage is 42.7% (Additional file 1).

Lastly, Model 4 in Tables 3 and 4 indicate that the economic capital of parents manifests a statistically significant association with depressive symptoms in the respondents over the capitals held by the respondents themselves for women but not for men.

## Discussion

We find that personal educational attainment and household income are both strongly and negatively related to depressive symptoms in our representative sample of South Korean adults. These findings are consistent with previous research in South Korea [39–41]. Plausible explanations for these associations are that higher education can foster intellectual and coping skills that serve as protective factors against depression [15, 18], high income reduces debilitating financial stressors that can contribute to the onset of depression [42] and well-paying jobs afford greater social prestige and better psychosocial and physical working conditions which in turn can be protective against depression [43].

We also find that a third or more of the association between the educational attainment of respondents and depressive symptoms is potentially mediated by household income. This is an example of what have been elsewhere referred to as capital conversions [44] or capital acquisition interplays [28], social processes whereby one form of capital facilitates the successful acquisition of another form of capital and consequently good health. In this case, institutionalized cultural capital in the form of educational credentials is presumably used by men and women to procure high-paying jobs (or spouses with high-paying jobs) and amass financial wealth which then mitigate the development of depressive symptoms.

In regards to intergenerational processes, we find that monetary inheritances or gifts from parents correspond with a lesser risk of depression for men. This is an example of what Veenstra and Abel [28] refer to as capital transmission processes, social processes whereby the capital of one party is transmitted to another person and then generates good health in the recipient. The absence of an association between inheritance and depressive symptoms among women may reflect the fact that monetary inheritances in South Korea tend to be much larger for sons than for daughters [45]. The largest transfer of financial assets from parents to children typically occurs when children marry. The parents of the groom often provide substantial economic support in the form of a house while the parents of the bride often provide household items. Unfortunately, our blunt measure of monetary inheritance does not allow us to empirically investigate the importance of the differential magnitude of inheritances for the mental health of men and women.

Finally, we find strong associations between parental educational capital and depressive symptoms among both men and women, associations that are almost entirely explained by the educational attainment of the adult children. In this case, we contend that parents strategically use their cultural capital to facilitate the acquisition of educational credentials by their children. In South Korea, educational qualifications virtually determine a person’s economic status in adulthood [46, 47] and, accordingly, South Korean parents are inclined to invest hugely in the education of their children. Our study indicates that this strategy tends to pay off in the positive mental health of their children later in life.

Our study has several limitations. First, the analysis utilizes cross-sectional data and as such causality cannot be confidently ascertained. Second, the measures of parental cultural capital and economic capital used in our study are based on respondent recall, an issue of measurement that may be especially problematic for older adults. Third, our measure of parental economic capital is a subjective judgement on the part of the adult children rather than an objective assessment of parental wealth. As a result we may have misrepresented, perhaps even underrepresented, the true nature of the association between parental economic capital and respondent depression. Fourth, our measure of monetary inheritance does not assess the magnitude of inheritances. Fifth, obtaining a degree from a prestigious university is an extremely important indicator of institutionalized cultural capital in South Korea. Unfortunately, the Korean Welfare Panel Study did not ask university educated survey respondents to identify the universities from which they obtained their degrees. In spite of these limitations, our study is the first, to our knowledge, that measures the parental economic capitals using economic conditions in childhood, monetary inheritance to investigate the intergenerational effects of the parental capitals on depressive symptoms in their adult children and the degree to which the capitals of the adult children explain them.

## Conclusions

Our study provides evidence for the intergenerational production of inequalities in depression in South Korea, findings that are consistent with studies conducted in the United States [12] and Finland [48]. Parental capitals are used to shape the accumulation of capitals by children, processes that militate against intergenerational mobility in South Korean society and ultimately produce inequalities in depression. In other words, socioeconomic inequalities in depression reflect a kind of social heredity in the accumulation of capitals in South Korean society.

## Additional file

Additional file 1: The KHB results. (DOCX 232 kb)

## Abbreviations

CES-D: Center for Epidemiological Studies Depression Scale
KHB decomposition: Karson/Holm/Breen decomposition
KHB method: Karson/Holm/Breen method

## Footnotes

1 In supplementary analyses, multinomial and ordered logistic regression models which distinguish between CES-D scores of 0, 1–8, and 9 or higher did not improve upon models that simply distinguish between CES-D scores of 0–8 and 9 or higher.
