# Supplementary material for: The intergenerational production of depression in South Korea: results from a cross-sectional study
Source: Int J Equity Health. 2017 Jan 13;16:13. doi: 10.1186/s12939-016-0513-7 (PMC5237188; doi:10.1186/s12939-016-0513-7)
Supplement: Additional file 1: — The KHB results. (DOCX 232 kb) [file 12939_2016_513_MOESM1_ESM.docx]

This table pertains to men. It describes the effects of parental education and economic conditions in childhood on depression, controlling for age, marital status and respondent education, and determines the degree to which these effects operate through and apart from respondent education. We note in the paper that the KHB decomposition indicates that 73.1% of the association between parental education (most versus least educated) and depression is caught up in the educational attainment of the men. This comes from the fact that 0.5101813 (the coefficient for least educated parents versus best educated parents before controlling for respondent education) – 0.1370812 (the coefficient for least educated parents versus best educated parents after controlling for respondent education) = 0.3731 and that 0.3712/0.5101813= 0.731. Economic conditions in childhood is not associated with depression before or after controlling for respondent education.

This table also pertains to men. It describes the effects of parental education and economic conditions in childhood on depression, controlling for age, marital status, respondent education, monetary inheritance and respondent income, and determines the degree to which these effects operate through and apart from respondent household income. Neither parental education nor economic conditions in childhood is significantly associated with depression before or after controlling for respondent income.

This table also pertains to men. It describes the effects of respondent education on depression, controlling for age, marital status, monetary inheritance, parental education, economic conditions in childhood and respondent income, and determines the degree to which this effect operates through and apart from respondent household income. In the paper we note that the KHB decomposition indicates that 44.7% of the association between respondent education (most versus least educated) and depression is caught up in the incomes of the respondents; this comes from the fact that 0.6777507/1.516885 = 0.447.

This table pertains to women. It describes the effects of parental education and economic conditions in childhood on depression, controlling for age, marital status and respondent education, and determines the degree to which these effects operate through and apart from respondent education. We note in the paper that the KHB decomposition indicates that 62.0% of the association between parental education (most versus least educated) and depression is caught up in the educational attainment of the women. This comes from the fact that 0.7902177 (the coefficient for least educated parents versus best educated parents before controlling for respondent education) – 0.300471 (the coefficient for least educated parents versus best educated parents after controlling for respondent education) = 0.4897467 and that 0.4897467/0.7902177= 0.620. We also note in the paper that “30.1% of the association between economic conditions (very poor versus rich or very rich) and depression is caught up in the educational attainment of the women (0.212265/0.7051945=0.301), but that economic conditions in childhood remains significantly associated with depression after controlling for respondent education (note that p=0.011 for the Diff row of 1.econchildhood2).

This table also pertains to women. It describes the effects of parental education and economic conditions in childhood on depression, controlling for age, marital status, respondent education, monetary inheritance and respondent income, and determines the degree to which these effects operate through and apart from respondent household income. Only economic conditions in childhood is significantly associated with depression before controlling for respondent income.

This table also pertains to women. It describes the effects of respondent education on depression, controlling for age, marital status, monetary inheritance, parental education, economic conditions in childhood and respondent income, and determines the degree to which this effect operates through and apart from respondent household income. In the paper we note that the KHB decomposition indicates that 42.7% of the association between respondent education (most versus least educated) and depression is caught up in the incomes of the respondents; this comes from the fact that 0.6269824/1.469859 = 0.427.
